# Association between periodontitis and the prevalence and prognosis of prediabetes: a population-based study

**DOI:** 10.1186/s12967-023-04340-y

**Published:** 2023-07-20

**Authors:** Liao Tan, Jie Liu, Zhaoya Liu

**Affiliations:** 1 Department of the Geriatrics, The Third Xiangya Hospital, Central South University, Changsha, 410013 Hunan China; 2 Department of Cardiology, The Third Xiangya Hospital, Central South University, Changsha, Hunan China; 3 Department of Cardiovascular Medicine, Xiangya Hospital, Central South University, Changsha, Hunan China

**Keywords:** Periodontitis, Prediabetes, NHANES

## Abstract

**Background:**

Diagnosis and intervention of prediabetes is an emerging method for preventing diabetic progression and complications. Periodontitis has been reported to strongly correlate with the dysregulation of glucose metabolism. Nonetheless, the relationship between periodontal status and the prevalence of prediabetes as well as its prognosis remains elusive. This study aimed to investigate the association of periodontitis with the prevalence of prediabetes and furtherly explore the all-cause mortality of different periodontal status among patients with prediabetes.

**Methods:**

The dateset from the National Health and Nutrition Examination Survey (NHANES) was utilized for our study. Participants were divided into two groups (with or without periodontitis) and further assigned into subgroups by different grades of periodontitis to analyze the association between periodontitis and prevalence of prediabetes. Then we analyzed the association between all-cause mortality and periodontitis among patients with prediabetes. Weighted multivariate logistic/Cox regression models were adopted in our study.

**Results:**

A total of 15390 participants were included and divided into a periodontitis group (n = 5033) and a nonperiodontitis group (n = 10357). The results showed that participants with periodontitis had a higher risk of prediabetes. After adjusting for covariables, more severe periodontitis was positively related to prediabetes (moderate vs. no periodontitis: OR = 1.46, 95% CI: 1.29–1.65; severe vs. no periodontitis: OR = 1.62, 95% CI 1.31–2.01). Furtherly, we explored the association between all-cause mortality and periodontal status among patients diagnosed with prediabetes (n = 4518) and found that severe (HR = 1.806, 95% CI 1.19–2.74) and moderate periodontitis (HR = 2.42, 95% CI 1.95–3.01) were associated with elevated all-cause mortality among patients with prediabetes.

**Conclusions:**

In general, the results suggest that periodontitis is positively associated with the prevalence and mortality of prediabetes. These results suggest that good management of periodontal status could be a potential strategy to reduce the occurrence and development of prediabetes.

**Supplementary Information:**

The online version contains supplementary material available at 10.1186/s12967-023-04340-y.

## Introduction

Prediabetes is the stage of intermediate hyperglycemia characterized by particular parameters, including impaired fasting glucose (IFG), impaired glucose tolerance (IGT) and specific scope of hemoglobin A1c (HbA1c) by The American Diabetes Association (ADA) [1]. Over the past few decades, the prevalence of prediabetes has been continuously increasing, with rates reaching as high as 43.5% in America [2]. Studies have shown that prediabetes patients have a higher risk of developing diabetes, cardiovascular complications and even death [3]. A recent study demonstrated that early management of prediabetes can alleviate the risk of progression to diabetes [4]. Despite this, attention and research toward prediabetes remains inadequate when compared to diabetes. Therefore, it is essential to identify more early interventions to prevent the progression of prediabetes to diabetes and cardiovascular complications.

As is commonly understood, individuals experiencing oral diseases are predisposed to metabolic dysregulation [5]. Periodontitis, a gradual and symptomless ailment, is a prevalent oral disease that results in tooth destruction and is generally irreversible [6]. The effects of periodontitis have been reported to be associated with cardiovascular complications in patients with dysglycemia, adversely impacting glycemic control [7]. A two-year follow-up study on the Indian population revealed that periodontitis was associated with an increased risk of poor glycemic control [8]. In addition, studies investigating the impact of periodontal treatment on glycemic control reported that a reduction in HbA1c of approximately 0.4% was found in diabetic patients with periodontal treatment [9]. Nevertheless, whether patients afflicted with periodontitis have a high risk of developing prediabetes and death has yet to be ascertained.

Currently, there are no effective methods to avert the onset of prediabetes. Hence, we attempted to find an early marker for prediabetes and intervene accordingly. Based on the findings of the association between periodontitis and glycemic control, we hypothesize that periodontitis is associated with a greater prevalence of prediabetes and its poor prognosis. Consequently, the purpose of this investigation is to ascertain whether periodontitis serves as a risk factor for the incidence and adverse outcomes of prediabetes, utilizing data from the National Health and Nutrition Examination Survey (NHANES).

## Methods and participants

### Study population

The NHANES (RRID:SCR_013201) is continuous survey research that provides population estimates related to the nutrition and health of adults and children in America. The survey employs a stratified, multistage probability design to recruit a representative sample of the American population. Data were obtained via personal structured interviews at home, health examinations at a mobile examination center, and specimen analyses in the laboratory. NHANES 1999–2004 and 2009–2014 were approved by The National Center for Health Statistics (NCHS) Ethics Review Board and conducted in accordance with the Helsinki Declaration of 1975, as revised in 2013. Informed consent was obtained from all participants. The acquisition and analysis of data was consistent with NHANES research requirements.

### Identification of prediabetes and periodontitis

Prediabetes was diagnosed by one of the following four principles: (1) Self-reported prediabetes: participants answer “Yes” to the question “Have you ever been told prediabetes/IGT/IFG/borderline diabetes/blood sugar higher than normal but not high enough to be called diabetes in diabetes questionnaire”. (2) HbA1c: 5.7%–7.0%. (3) Fasting plasma glucose: 5.6–7.0 mmol/L. (4) oral glucose tolerance test: 7.8–11.0 mmol/L [10].

Periodontitis was defined as clinical attachment loss (CAL; the difference between pocket depth and gingival recession) ≥ 3 mm. This has been recommended for epidemiological studies as an extent measure of periodontitis and is henceforth referred to as CAL extent. The severity of periodontitis was defined by following Centers for Disease Control and Prevention in partnership with the American Academy of Periodontology (CDC–AAP) case definitions for surveillance of periodontitis [11].

### Inclusion and exclusion process

**Fig. 1:**
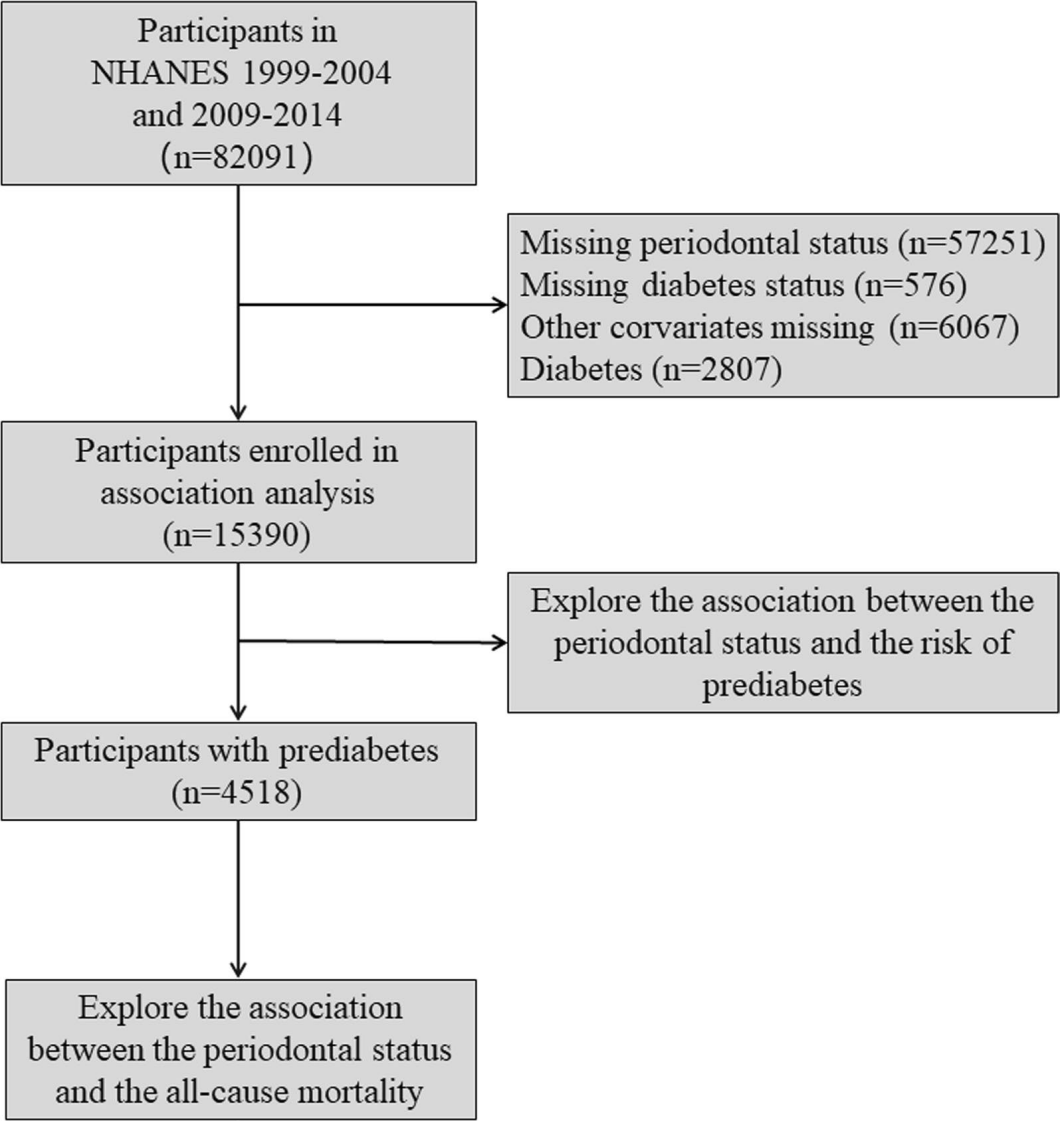
Study workflow

We used the data from the NHANES 1999–2004 and 2009–2014 cycles. First, 15390 patients with clear periodontal, prediabetes and other covariable data were included to explore the association between periodontitis status and the risk of prediabetes. Furthermore, we enrolled 4518 participants with prediabetes of 15390 sample to further explore the association between periodontitis status and all-cause mortality (Fig. 1).

### Outcomes

The outcome of association analysis was diagnosed with prediabetes. The outcome of survival analysis was all-cause mortality with periodontitis or not among patients diagnosed with prediabetes. All-cause mortality was ascertained by linkage to National Death Index records through 2018. ICD-10 was used to determine the causes of death.

### Assessment of covariates

Standardized questionnaires obtained information on sociodemographic characteristics, smoking status, and alcohol consumption. Never smokers were classified as those who reported smoking < 100 cigarettes during their lifetime. Those who smoked > 100 cigarettes in their lifetime were considered current smokers, and those who smoked > 100 cigarettes and had quit smoking were considered former smokers. Nondrinkers were defined as consuming no drinks per year. Heavy alcohol use was defined as ≥ 3 drinks per day for females, ≥ 4 drinks per day for males, or binge drinking [≥ 4 drinks on the same occasion for females, ≥ 5 drinks on the same occasion for males] on 5 or more days per month. Moderate alcohol use was defined as ≥ 2 drinks per day for females, ≥ 3 drinks per day for males, or binge drinking ≥ 2 days per month. Other frequencies of alcohol consumption were defined as mild alcohol use. Body mass index (BMI) was calculated from weight/height^2^ (kg/m^2^). Diabetes was defined as follows: (1) doctor diagnosed you as having diabetes; (2) HbA1c > 7%; (3) fasting glucose ≥ 7.0 mmol/L; (4) random blood glucose ≥ 11.1 mmol/L, (5) two-hour oral glucose tolerance test (OGTT) blood glucose ≥ 11.1 mmol/L; and (5) use of diabetes medication or insulin. Hyperlipidemia was defined as follows: (1) triglyceride ≥ 150 mg/dL, (2) cholesterol ≥ 200 mg/dL, (3) low-density lipoprotein (LDL)-cholesterol ≥ 130 mg/dL, (4) high-density lipoprotein (HDL)-cholesterol < 40 mg/dL (male); 50 mg/dL (female), and (5) use of hyperlipidemia medication. Coronary heart disease (CHD) was defined as self-reported CHD. Homeostasis model assessment-insulin resistance (HOMA-IR) was calculated by the following formula: HOMA-IR = [fasting plasma glucose (mU/L) × fasting plasma glucose (mmol/L)]/22.5. Homeostasis model assessment-insulin sensitivity (HOMA-IS) was estimated by the following formula: HOMA-IS = 22.5/[fasting plasma glucose (mU/L) × fasting plasma glucose (mmol/L)]. In addition, strict laboratory analyses were performed, including the assessment of glucose, insulin, HbA1c, triglyceride, HDL, LDL, and C-reactive protein (CRP) levels at baseline. Further details of these measurements were documented in the NHANES Laboratory Medical Technologists Procedures Manual.

### Statistical analysis

All analyses incorporated sample weights, strata, and primary sampling units to produce accurate national estimates. Sample characteristics are reported as the means (SEs) for normally distributed continuous variables, medians (interquartile ranges) for nonnormally distributed continuous variables, and percentages for categorical variables. Weighted multivariate logistic regression models were performed to estimate the OR and 95% confidence interval associated with periodontitis status and prediabetes with the first group as a reference. Weighted Cox proportional hazards regression was used to estimate hazard ratios (HRs) and 95% CIs of all-cause mortality in relation to periodontitis. Person-time was calculated as the interval between the NHANES interview date and the date of death or the end of the follow-up (31-December-2018), whichever occurred first. We constructed three statistical models. Model 1: no covariates were adjusted. Model 2: Age (continuous), gender (male or female), race/ethnicity (Mexican American, other Hispanic, non-Hispanic white, non-Hispanic black, or other Race), and education level (less than high school, high school, more than high school) were adjusted. Model 3: age (continuous), gender (male or female) and race/ethnicity (Mexican American, other Hispanic, non-Hispanic white, non-Hispanic black, or other Race), education level (less than high school, high school, more than high school), smoking status (never, former, or current), alcohol use (never, former, mild, moderate. severe), BMI (continuous), hypertension (no or yes), hyperlipidemia (yes or no), and CHD (yes or no) were adjusted. Any individuals with missing covariate data were not included in the analyses in Model 2 and Model 3 (listwise deletion). Furthermore, stratified analyses were conducted to examine whether the detected association differed by age, sex, and body mass index. All analyses were performed using R software (4.1.0). Two-sided P < 0.05 was considered statistically significant. The Bonferroni correction threshold was used to account for multiple comparisons.

## Results

### Participant characteristics

In this study, 15390 participants were enrolled in our analysis. There were 5033 participants with periodontitis and 10357 participants without periodontitis. Compared to participants without periodontitis, participants with periodontitis were more likely to be female, older, have a higher education level, hypertension, hyperlipidemia, CHD, be former alcohol user, smoke, have a higher BMI and glucose/insulin/HbA1c/HOMA-IR, have prediabetes, and have diabetes (Table 1).

**Table 1:** Baseline characteristics of included participants in NHANES 1999–2004 and 2009–2014

Variables	Total − 15390	Without Periodontitis − 10357	With Periodontitis − 5033	P value
Age, years	49.79 ± 0.22	43.26 ± 0.24	54.05 ± 0.29	< 0.001
Gender, %				< 0.001
Female	7840 (50.94%)	4794 (46.98%)	3046 (59.33%)	
Male	7550 (49.06%)	5563 (53.02%)	1987 (40.67%)	
Race, %				< 0.001
Non-Hispanic White	7493 (48.69%)	5342 (74.39%)	2151(66.38%)	
Non-Hispanic Black	2872 (18.66%)	1752 (8.60%)	1120 (12.25%)	
Mexican American	2810 (18.26%)	1940 (7.16%)	870 (8.85%)	
Other Race	1108 (7.20%)	656 (4.55%)	452 (6.82%)	
Other Hispanic	1107 (7.19%)	667 (5.31%)	440 (5.71%)	
Education level, %				< 0.001
Less than high school	1540 (10.01%)	848 (3.44%)	692 (7.73%)	
High school	5624 (36.54%)	3515 (31.03%)	2109 (40.36%)	
More than high school	8226 (53.45%)	5994 (65.54%)	2232 (51.91%)	
Prediabetes, %				< 0.001
No	10872 (70.64%)	7910 (79.65%)	2962 (62.28%)	
Yes	4518 (29.36%)	2447 (20.35%)	2071 (37.72%)	
Hypertension, %				
No	9748 (63.34%)	7102 (71.11%)	2646 (56.34%)	< 0.001
Yes	5642 (36.66%)	3255 (28.89%)	2387 (43.66%)	
Hyperlipidemia, %				
No	4549 (29.56%)	3203 (30.88%)	1346 (25.83%)	< 0.001
Yes	10841 (70.44%)	7154 (69.12%)	3687 (74.17%)	
CHD, %				
No	15105 (98.15%)	10235 (99.09%)	4870 (96.73%)	< 0.001
Yes	285 (1.85%)	122 (0.91%)	163 (3.27%)	
Alcohol use, %				< 0.001
Never	1951 (12.68%)	1334 (10.73%)	617 (9.41%)	
Former	2384 (15.49%)	1383 (11.57%)	1001 (17.97%)	
Mild	5486 (35.65%)	3757 (38.53%)	1729 (36.16%)	
Moderate	2348 (15.26%)	1737 (18.30%)	611 (13.89%)	
Severe	3221 (20.93%)	2146 (20.87%)	1075 (22.57%)	
MET, min/week	7249.87 ± 227.66	7265.08 ± 231.98	7094.30 ± 463.56	0.71
Smoke status, %				< 0.001
Never	8407 (54.63%)	6163 (58.14%)	2244 (43.16%)	
Former	3662 (23.79%)	2256 (22.76%)	1406 (28.39%)	
Now	3321 (21.58%)	1938 (19.10%)	1383 (28.45%)	
BMI	28.07 ± 0.07	27.97 ± 0.09	28.33 ± 0.14	0.03
Triglyceride, mg/dL	131.66 ± 1.74	131.56 ± 2.22	131.96 ± 3.00	0.92
Cholesterol, mg/dL	200.80 ± 0.51	199.87 ± 0.56	203.43 ± 0.85	< 0.001
HDL, mg/dL	53.32 ± 0.21	53.26 ± 0.24	53.48 ± 0.40	0.63
LDL, mg/dL	120.70 ± 0.55	119.93 ± 0.63	122.91 ± 0.97	0.01
Glucose, mg/dL	96.34 ± 0.19	95.476 ± 0.22	98.89 ± 0.30	< 0.001
Insulin, uu/mL	11.13 ± 0.16	10.97 ± 0.17	11.57 ± 0.25	0.02
HbA1c	5.34 ± 0.01	5.29 ± 0.01	5.48 ± 0.01	< 0.001
HOMA-IR	2.71 ± 0.04	2.65 ± 0.05	2.88 ± 0.07	0.001
HOMA-IS	0.61 ± 0.01	0.62 ± 0.01	0.58 ± 0.02	0.06
CRP, mg/dL	0.36 ± 0.01	0.35 ± 0.01	0.37 ± 0.01	0.22

*CHD* Coronary Heart Disease, *MET* Metabolic Equivalent of Task, *BMI* Body Mass Index, *HbA1C* Glycated hemoglobin A1, *HOMA-IR* Homeostasis Model Assessment for Insulin Resistance, *HOMA-IS* Homeostasis Model Assessment-Insulin Sensitivity

### Logistic regression between periodontitis and prediabetes

We conducted weighted multivariate logistic regression models to explore the association between periodontitis status and prediabetes (Table 2). Compared to nonperiodontitis, periodontitis was positively associated with prediabetes in all models (OR = 2.37, 95% CI 2.16–2.60; 1.45, 95% CI 1.30–1.61; 1.47, 95% CI 1.31–1.63). The p value of all models was < 0.001. We further stratified periodontitis into three statuses according to the CDC–AAP case definitions. In Model 1, all periodontitis statuses were significantly associated with prediabetes (OR = 1.42, 95% CI 1.15–1.77; 2.39, 95% CI 2.15–2.66; 2.74, 95% CI 2.26–3.32). The p value of all periodontitis statuses equaled 0.001. After adjusting for covariables, moderate and severe periodontitis were positively associated with prediabetes in Model 2 (OR = 1.46, 95% CI 1.30–1.65, 1.57, 95% CI 1.27–1.94) and Model 3 (OR = 1.46, 95% CI 1.29–1.65; 1.62, 95% CI 1.31–2.01). The p value of moderate and severe periodontitis was < 0.001 in Models 2 and 3. Furthermore, a significant association remained between periodontitis and prediabetes in most subgroups (Additional file 1: Table S1).

**Table 2:** Association between the pre-diabetes and periodontitis among participants in NHANES 1999–2014

Periodontal status	Model 1	Model 2	Model 3
	OR (95% CI)	OR (95% CI)	OR (95% CI)
	P value	P value	P value
Periodontitis			
No	1	1	1
Yes	2.37 (2.16,2.60)	1.45 (1.30,1.61)	1.47 (1.31,1.63)
	< 0.001	< 0.001	< 0.001
Periodontitis by CDC-AAP			
No	1	1	1
Mild	1.42 (1.15,1.77)	1.20 (0.96, 1.51)	1.12 (0.89,1.41)
	0.001	0.111	0.325
Moderate	2.39 (2.15,2.66)	1.46 (1.30, 1.65)	1.46 (1.29,1.65)
	0.001	< 0.001	< 0.001
Severe	2.74 (2.26,3.32)	1.57 (1.27, 1.94)	1.62 (1.31, 2.01)
	0.001	< 0.001	< 0.001

Model 1: no covariates were adjusted. Model 2: age (continuous), gender (male or female) and race/ethnicity (Mexican American, other Hispanic, non-Hispanic white, non-Hispanic black, or other Race), education level (less than high school, high school, more than high school) were adjusted. Model 3: age (continuous), gender (male or female) and race/ethnicity (Mexican American, other Hispanic, non-Hispanic white, non-Hispanic black, or other Race), education level (less than high school, high school, more than high school), smoking status (never, former, or current), alcohol use (never, former, mild, moderate. severe), BMI (continuous), hypertension (no or yes), hyperlipidemia (yes or no), CHD (yes or no) were adjusted

*CDC-AAP* Disease Control and Prevention-American Academy of Periodontology, *BMI* Body Mass Index, *CHD* Coronary Heart Disease

### Cox regression between periodontitis and mortality of patients with prediabetes

**Fig. 2:**
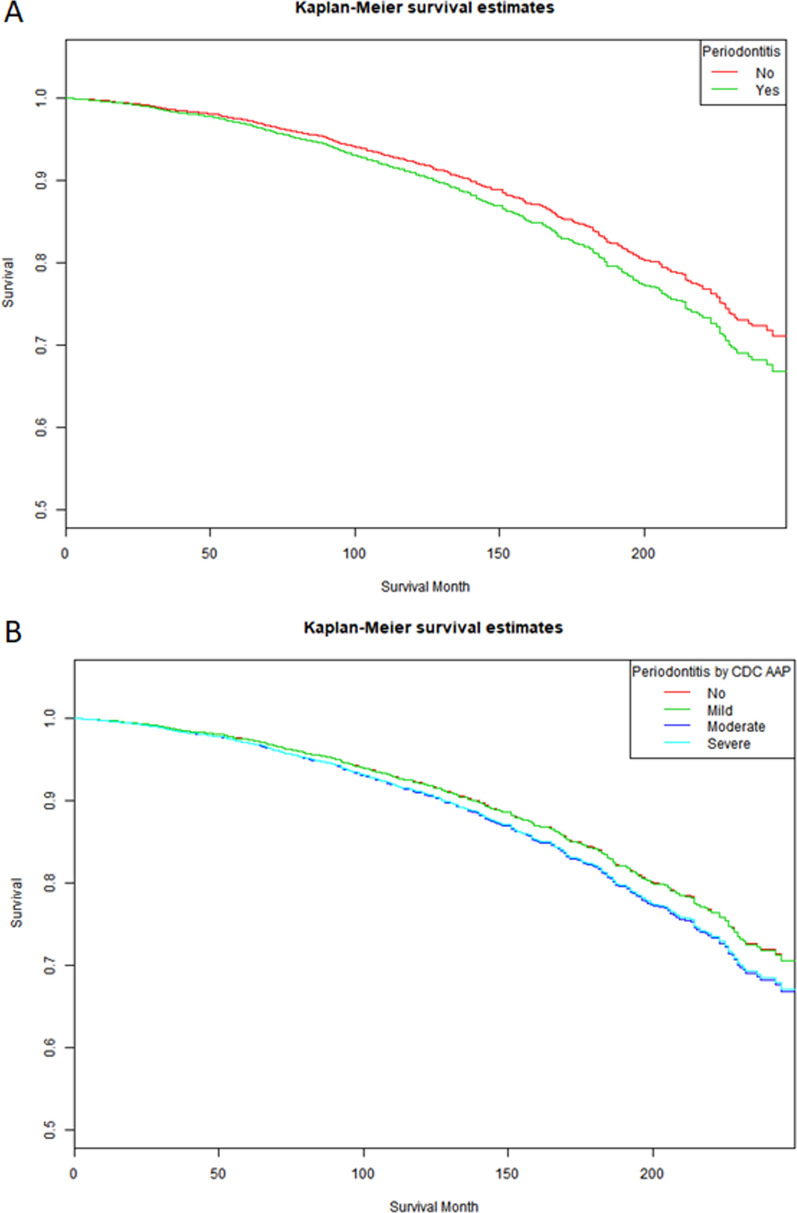
Kaplan‒Meier survival curve of prediabetic patients with/without periodontitis. **A**. Kaplan‒Meier survival curve of the prediabetes patients with/without periodontitis; **B**. Kaplan‒Meier survival curve of the prediabetes patients with different statuses of periodontitis

A total of 4518 participants with prediabetes were included to further explore the correlation between periodontitis status and diabetes mortality by weighted Cox proportional hazards regression. The characteristics of these participants are shown in Additional file 1: Table S2. The average follow-up time was 128.41 ± 1.67 months (Additional file 1: Table S2). Compared to the nonperiodontitis group, participants with periodontitis were pivotally correlated with mortality of prediabetes in all models (HR = 2.41, 95% Cl 1.98–2.94, p < 0.001; 1.47, 95% Cl 1.20–1.80, p < 0.001; 1.28, 95% Cl 1.04–1.57, p = 0.02). After stratifying the status of periodontitis, moderate and severe periodontitis were positively correlated with mortality of prediabetes (HR = 2.42, 95% Cl 1.95–3.01, p < 0.001; 1.806, 95% Cl 1.19–2.74, p = 0.005). After further adjustments for age, sex, race, education level, alcohol use, BMI, hypertension, CHD, moderate periodontitis was significantly associated with mortality of prediabetes in Model 2 (HR = 1.45, 95% CI 1.17–1.80, p < 0.001) and Model 3 (HR = 1.25, 95% CI 1.00–1.55, p = 0.047) (Table 3). The Kaplan–Meier survival curve presented similar results (Fig. 2). In the subgroup analysis, older age, female sex, high education level, mild alcohol use, former smoking, hypertension, hyperlipidemia, and higher BMI were positively correlated with all-cause mortality of prediabetes (Additional file 1: Table S3).

**Table 3:** All-cause mortality of patients with pre-diabetes during different periodontal status in NHANES 1999–2004 and 2009–2014

Periodontal status	Model 1	Model 2	Model 3
	HR (95% CI)	HR (95% CI)	HR (95% CI)
	P value	P value	P value
Periodontitis			
No	1	1	1
Yes	2.41 (1.98,2.94)	1.47 (1.20,1.80)	1.28 (1.04,1.57)
	< 0.001	< 0.001	0.02
Periodontitis by CDC-AAP			
No	1	1	1
Mild	0.98 (0.59,1.64)	0.91 (0.54, 1.51)	0.91 (0.54,1.51)
	0.944	0.705	0.705
Moderate	2.42 (1.95,3.01)	1.45 (1.17, 1.80)	1.25 (1.00,1.55)
	< 0.001	< 0.001	0.047
Severe	1.806 (1.19,2.74)	1.20 (0.71, 2.02)	1.02 (0.64, 1.65)
	0.005	0.495	0.912

Model 1: no covariates were adjusted. Model 2: age (continuous), gender (male or female) and race/ethnicity (Mexican American, other Hispanic, non-Hispanic white, non-Hispanic black, or other Race), education level (less than high school, high school, more than high school) were adjusted. Model 3: age (continuous), gender (male or female) and race/ethnicity (Mexican American, other Hispanic, non-Hispanic white, non-Hispanic black, or other Race), education level (less than high school, high school, more than high school), smoking status (never, former, or current), alcohol use (never, former, mild, moderate. severe), BMI (continuous), hypertension (no or yes), hyperlipidemia (yes or no), CHD (yes or no) were adjusted

*CDC-AAP* Disease Control and Prevention-American Academy of Periodontology, *BMI* Body Mass Index, *CHD* Coronary Heart Disease

## Discussion

With the discovery of pathophysiological processes, periodontitis is regarded as a chronic inflammatory disease that is associated with other noninfected systemic diseases, including cardiovascular and metabolic diseases, rheumatic arthritis, and Alzheimer’s disease [12]. Chiu [13], Demmer [14], and Saito [15] et al. reported that periodontitis was significantly associated with the elevation of HbA1c and the development of insulin tolerance. To further explore this relationship, our study aimed to investigate the association between periodontitis and prediabetes.

The findings of this study indicate a significant association between moderate and severe periodontitis and the occurrence of prediabetes. Similarly, Desvarieux [16] and Shiva [17] et al. reported that periodontal infection and inflammation were associated with insulin resistance and impaired glucose tolerance in the nationally representative American and Iranian sample. Nonetheless, Pérez [18] et al. established a 3-year oral health status cohort enrolled overweight adults and no association between periodontitis and the risk of prediabetes/diabetes were reported. But the median follow-up time of this longitudinal study was 2.96 years which may be too short to influence the results of the prevalence risk of prediabetes/diabetes. And in this study, patients with moderate or severe periodontitis had a significant improvement in periodontal health during follow-up, which could have a great influence on the results. Simultaneously, Amar [19] and Loos [20] et al. demonstrate that severe periodontitis was associated with the elevation of HbA1c and HOMA-IR. We found that moderate periodontitis was also positively associated with prediabetes. On the one hand, this may be due to the uniform quality control of periodontal examinations in the NHANES study. On the other hand, we utilized the CDC–AAP criterion to define the status of periodontitis. This criterion can effectively promote the clinical periodontal condition. But it excluded buccal and/or lingual sites which can impact periodontal condition. Therefore, large-scale prospective studies are still needed to investigate the impact of periodontitis on the development and prognosis of prediabetes.

Previous studies just suggested severe periodontitis was significantly associated with mortality of type 2 diabetes mellitus [21]. In our study, we found that moderate periodontitis was found to be positively correlated with all-cause mortality in individuals with prediabetes. We assumed that chronic systemic inflammation aroused by periodontitis and blood sugar dysregulation promotes the progression of various systemic diseases, including cardiovascular diseases, resulting in increased mortality [22].

Although abundant research has revolved around periodontitis and IGT/insulin resistance, the mechanism remains unclear. A recent study demonstrated that the oral microbiota contained approximately 500–700 prevalent taxa [23]. Among them, Porphyromonas gingivalis (P. gingivalis) and *Aggregatibacter actinomycetemcomitans* (*A. actinomycetemcomitans*) were shown to be classical pathogens of periodontitis [24]. These pathogens activate the innate immune system in situ and secrete inflammatory factors [25]. Several studies have demonstrated that gingival crevicular fluid (GCF) and serum of patients with periodontitis contain high levels of TNF-α, IL-1β, IL-6, and other proinflammatory factors that play a pivotal role in the development of insulin secretion and resistance [26, 27]. Izumi [28] et al. built a periodontitis animal model affected by *A. actinomycetemcomitans* and performed an insulin/glucose tolerance test. The level of insulin resistance was significantly elevated. Furthermore, Luan et al. utilized *P. gingivalis* to establish an animal model [29]. They revealed that the levels of TNF-α and IL-6 were increased and the expression of PPARa, Irs1, and sitr1 was decreased, which suggested that glucose metabolism was dysregulated. Regarding clinical trials, Kocher [30] et al. conducted a multiple-center randomized study to explore the influence of nonsurgical periodontal treatment on prediabetes status. They revealed that treatment of periodontitis decreased the levels of HbA1c and CRP, which was consistent with our results. Kowall [31] et al. implied that periodontitis was only associated with poorly controlled diabetes, not prediabetes. The reason for the different conclusions may be the different races of the included participants and the different definitions of periodontitis. Therefore, further long-term follow-up is needed to clarify the role of root canal treatment of severe periodontitis in improving prediabetes.

In addition, these pathogens produce abundant virulence factors directly and via outer membrane vesicles (OMVs). These virulence factors, which activate macrophages and induce systemic inflammation, included lipopolysaccharide (LPS), protein, lipid, muramic acid, RNA, and DNA [32–34]. Subcutaneous chronic infusion of *P.* gingivalis LPS into high-fat diet-fed mouse enhanced insulin resistance of mouse [35]. Moreover, Ruiz [36] and Ivanovski [37] et al. noticed that GCF of patients with periodontitis had elevated concentrations of extracellular vesicles, especially OMV. OMV is a 50–250 nm particle derived from gram-negative bacteria [38]. OMV from *P. gingivalis* increased the expression of Toll-like receptors and nod-like receptors, which mediated insulin resistance signaling [38, 39]. Nonetheless, animal experiments and clinical trials to clarify the mechanism of virulence factors of these key pathogens in prediabetes are still lacking.

## Clinical implication

The early diagnosis and intervention of prediabetes are important for preventing diabetic conversion and complications. Currently, the first-line therapy for prediabetes was lifestyle modification or metformin. Intensive lifestyle modification was more beneficial than metformin, such as diet, exercise, and weight loss [40]. However, there are no effective methods to decrease the morbidity of prediabetes. Our findings offer valuable insights into elucidating the association between periodontitis and prediabetes. As periodontitis is a common disease in the adult population, our findings provide a novel insight into the risk and treatment of prediabetes from the perspective of oral health status. Endocrinologists should pay attention to the oral condition of patients with prediabetes rather than only diabetes patients. Endocrinologists should also focus not only on oral health during the treatment of diabetes but also pay attention to the prevention of the development and deterioration of prediabetes through oral health management. Future studies are necessary to investigate whether therapeutic interventions aimed at addressing periodontitis can reverse or impede the progression of prediabetes to diabetes and thereby prevent subsequent negative health outcomes.

## Limitations

This study explored the association between periodontitis and prediabetes. We conducted a large-scale sample retrospective study and adjusted for potential covariables to guarantee validity. However, some limitations remain. First, we only enrolled American adults in our study, which does not represent the population of another region. Second, although we considered every confounding variable, some variables still affected the prevalence and mortality of prediabetes. Third, the accuracy of periodontal pocket detection is limited by the type of probe. Therefore, the classification of periodontitis severity should be further demonstrated. We will conduct a further multicenter study and animal experiment to further validate our findings.

## Conclusion

In summary, moderate and severe periodontitis were significantly associated with the risk of prediabetes. Moderate periodontitis was positively correlated with mortality in prediabetes patients. This suggests that periodontitis may be a new and valuable risk indicator of prediabetes. Early treatment of periodontitis may improve the prognosis of prediabetes.

## Supplementary Information

**Additional file 1:** **Supplementary tables: Table S1**. Subgroup analyses of the associations between periodontitis and the risk of prediabetes. **Table S2**. Baseline characteristics of included participants with prediabetes in NHANES 1999–2004 and 2009-2014. **Table S3**. Subgroup analyses of the associations between periodontitis and the mortality of prediabetes.

## Data Availability

The data used in this study are from a public database at https://www.cdc.gov/nchs/nhanes/index.htm, which can be accessed by everyone through the links provided in the paper.

## Abbreviations

DM: Diabetes mellitus
NHANES: National Health and Nutrition Examination Survey
NCHS: National Center for Health Statistics
CAL: Clinical Attachment Loss
CDC–AAP: Centers for Disease Control and Prevention in partnership with the American Academy of Periodontology
BMI: Body Mass Index
OGTT: Oral glucose tolerance test
LDL: Low-Density Lipoprotein
HDL: High-Density Lipoprotein
CHD: Coronary Heart Disease
HOMA-IR: Homeostasis Model Assessment-Insulin Resistance
HOMA-IS: Homeostasis Model Assessment-Insulin Sensitivity
CRP: C-Reactive Protein
HR: Hazard Ratio
P. gingivalis: Porphyromonas gingivalis
A. actinomycetemcomitans: Aggregatibacter actinomycetemcomitans
GCF: Gingival Crevicular Fluid
OMV: Outer membrane vesicles
LPS: Lipopolysaccharide
