# Supplementary material for: Association between periodontitis and the prevalence and prognosis of prediabetes: a population-based study
Source: J Transl Med. 2023 Jul 20;21:484. doi: 10.1186/s12967-023-04340-y (PMC10357600; doi:10.1186/s12967-023-04340-y)
Supplement: Supplementary file 1 — Additional file 1: Supplementary tables: Table S1. Subgroup analyses of the associations between periodontitis and the risk of prediabetes. Table S2. Baseline characteristics of included participants with prediabetes in NHANES 1999–2004 and 2009-2014. Table S3. Subgroup analyses of the associations between periodontitis and the mortality of prediabetes. [file 12967_2023_4340_MOESM1_ESM.docx]

**Association between periodontitis and the prevalence and prognosis of prediabetes: a population-based study**

Liao Tan MD.^1,2^, Jie Liu MD.^3^, Zhaoya Liu MD. Ph.D^1,2^

1. Department of the Geriatrics, The Third Xiangya Hospital, Central South University, Changsha, Hunan, China ;

2. Department of Cardiology, The Third Xiangya Hospital, Central South University, Changsha, Hunan, China

3. Department of Cardiovascular Medicine, Xiangya Hospital, Central South University, Changsha, Hunan, China

**Running title:** Periodontitis and prediabetes

**Corresponding Author’s Contact Information**

Zhaoya Liu MD. Ph.D, Department of the Geriatrics, The Third Xiangya Hospital, 138 Tongzipo Road, Changsha, Hunan Province, 410013, China. Tel: 86-15243607431 E-mail: [liuzhaoya@csu.edu.cn](mailto:liuzhaoya@csu.edu.cn) ORCID：0000-0002-0964-8781

**Number of references:** 33

**Number of tables:** 3

**Number of figures:** 2

**Word length for the article’s text:** 2926

**Word length for the abstract:** 298

**Keywords:** periodontitis, prediabetes, NHANES

**Supplementary Table 1: Subgroup analyses of the associations between periodontitis and the risk of prediabetes.**

| **Variables** | **n** | **Without Periodontitis** | **With Periodontitis** |
| --- | --- | --- | --- |
|  |  | **OR 95% Cl P-value** | **OR 95% Cl P-value** |
| Age, years  ≥20, <45  ≥45, <65  ≥65  Gender, %  Female  Male  Race, %  Non-Hispanic White  Non-Hispanic Black  Mexican American  Other Race  Other Hispanic  Education level, %  Less than high school  High school  More than high school  Alcohol use, %  Never  Former  Mild  Moderate  Severe  Smoking, %  Never  Former  Now  Hypertension, %  No  Yes  Hyperlipidemia, %  No  Yes  BMI, kg/m^2^  <24.9  ≥24.9, <29.9  ≥29.9 | 7124  5569  2697  7840  7550  7493  2872  2819  1108  1107  1540  5624  8226  1951  2384  5486  2348  3221  8407  3662  3321  9748  5642  4595  10841  4841  5537  5012 | Reference  Reference  Reference  Reference  Reference  Reference  Reference  Reference  Reference  Reference  Reference  Reference  Reference  Reference  Reference  Reference  Reference  Reference  Reference  Reference  Reference  Reference  Reference  Reference  Reference  Reference  Reference  Reference | 1.45 (1.17, 1.81) <0.001  1.52 (1.31, 1.77) <0.001  1.43 (1.19, 1.73) <0.001  1.34 (1.16, 1.54) <0.001  1.79 (1.55, 2.06) <0.001  1.48 (1.28, 1.72) <0.001  1.32 (1.05, 1.65) 0.02  1.45 (1.12, 1.89) 0.01  1.71 (1.12, 2.61) 0.01  1.26 (0.91, 1.75) 0.17  1.52 (1.09, 2.10) 0.01  1.77 (1.44, 2.18) <0.001  1.33 (1.14, 1.56) <0.001  1.42 (1.02, 1.96) 0.04  1.60 (1.28, 2.00) <0.001  1.47 (1.22, 1.78) <0.001  1.42 (0.99, 2.05) 0.06  1.55 (1.19, 2.03) 0.002  1.25 (1.07, 1.47) 0.01  1.50 (1.21, 1.88) <0.001  2.12 (1.62, 2.76) <0.001  1.55 (1.33, 1.80) <0.001  1.45 (1.23, 1.71) <0.001  1.63 (1.27, 2.10) <0.001  1.48 (1.31, 1.67) <0.01  2.05 (1.73, 2.44) <0.001  1.48 (1.24, 1.78) <0.001  1.33 (1.12, 1.57) 0.001 |

BMI: Body mass index; CHD: Chronic heart disease

**Supplementary Table 2: Baseline characteristics of included participants with prediabetes in NHANES 1999–2004 and 2009-2014.**

| **Variables** | **Total**  **(4518)** | | **Without Periodontitis**  **(2447)** | **With Periodontitis**  **(2071)** | **P value** |
| --- | --- | --- | --- | --- | --- |
| Age, years  Gender, %  Female  Male  Race, %  Non-Hispanic White  Non-Hispanic Black  Mexican American  Other Race  Other Hispanic  Education level, %  Less than high school  High school  More than high school  Mortality, %  No  Yes  Hypertension, %  No  Yes  Hyperlipidemia, %  No  Yes  CHD，%  No  Yes  Alcohol use, %  Never  Former  Mild  Moderate  Severe  MET,min/week  Smoke status, %  Never  Former  Now  BMI, kg/m^2^  Triglyceride, mg/dL  Cholesterol, mg/dL  HDL, mg/dL  LDL, mg/dL  Glucose, mg/dL  Insulin, uu/mL  HbA1c  HOMA-IR  HOMA-IS  CRP, mg/dL | | 52.78±0.28  2490 (55.11%)  2028 (44.89%)  1963 (43.45%)  967 (21.4%)  834 (18.46%)  395 (8.74%)  359 (7.95%)  559 (12.37%)  1736 (38.42%)  2223 (49.2%)  3845 (85.10%)  673 (14.90%)  2240 (49,58%)  2278 (50.42%)  850 (18.81%)  3668 (81.19%)  4377 (96.88%)  141 (3.12%)  627 (13.88%)  867 (19.19%)  1646 (36.43%)  554 (12.26%)  824 (18.24%)  5883.35±365.51  2377 (52.61%)  1235 (27.34%)  906 (20.05%)  30.06±0.15  150.63±3.29  204.44±0.92  50.44±0.34  124.60±1.02  105.17±0.21  13.97±0.27  5.65±0.011  3.67±0.08  0.42±0.01  0.50±0.02 | 50.01±0.33  1231 (52.40%)  1216 (47.60%)  1126 (71.54%)  484 (10.48%)  465 (7.57%)  193 (5.22%)  179 (5.19%)  246 (4.32%)  845 (32.64%)  1356 (63.04%)  2117 (89.88%)  330 (10.12%)  1306 (55.34%)  1141 (44.66%)  456 (16.96%)  1991 (83.04%)  2398 (98.09%)  49 (1.91%)  355 (11.95%)  405 (15.16%)  924 (41.10%)  335 (15.41%)  428 (16.38%)  5860.59±383.98  1470 (59.87%)  615 (26.24%)  362 (13.89%)  30.51±0.19  155.18±4.75  205.04±1.18  49.97±0.43  124.50±1.24  105.23±0.22  14.34±0.34  5.60±0.01  3.77±0.10  0.40±0.01  0.53±0.03 | 57.00±0.41  1259 (58.91%)  812 (41.09%)  837 (64.55%)  483 (12.85%)  369 (9.51%)  202 (7.66%)  180 (5.44%)  313 (8.68%)  891 (43.25%)  867 (48.06%)  1728 (84.68%)  343 (15.32%)  934 (48.58%)  1137 (51.42%)  394 (18.93%)  1677 (81.07%)  1979 (95.22%)  92 (4.78%)  272 (10.11%)  462 (20.71%)  722 (36.80%)  219 (12.68%)  396 (19.69%)  6011.32±867.59  907 (41.70%)  620 (30.23%)  544 (28.07%)  29.38±0.22  142.75±4.59  203.52±1.25  51.15±0.61  124.77±1.40  105.05±0.35  13.31±0.35  5.73±0.01  3.49±0.09  0.45±0.01  0.44±0.02 | <0.001  <0.001  0.003  <0.001  <0.001  <0.001  0.22  <0.001  <0.001  0.87  <0.001  <0.001  0.08  0.34  0.13  0.87  0.61  0.02  <0.001  0.02  0.002  0.04 |

CHD: Coronary Heart Disease; MET: Metabolic Equivalent of Task; BMI: Body Mass Index; Hba1C: Glycated hemoglobin A1; HOMA-IR: Homeostasis Model Assessment for Insulin Resistance; HOMA-IS: Homeostasis Model Assessment-Insulin Sensitivity

**Supplementary table 3: Subgroup analyses of the associations between periodontitis and the mortality of prediabetes.**

| **Variables** | **n** | **Number of Death** | **Without Periodontitis** | **With Periodontitis** |
| --- | --- | --- | --- | --- |
|  |  |  | **HR 95% Cl P-value** | **HR 95% Cl P-value** |
| Age, years  ≥20, <45  ≥45, <65  ≥65  Gender, %  Female  Male  Race, %  Non-Hispanic White  Non-Hispanic Black  Mexican American  Other Race  Other Hispanic  Education level, %  Less than high school  High school  More than high school  Alcohol use, %  Never  Former  Mild  Moderate  Severe  Smoking, %  Never  Former  Now  Hypertension, %  No  Yes  Hyperlipidemia, %  No  Yes  BMI, kg/m^2^  <24.9  ≥24.9, <29.9  ≥29.9 | 1257  2041  1220  2490  2028  1963  967  834  395  359  559  1736  2223  627  867  1646  554  824  2377  1235  906  2240  2278  850  3668  943  1641  1934 | 35  177  461  409  264  422  113  95  18  25  115  274  284  99  185  254  57  78  295  252  126  210  463  126  547  187  262  224 | Reference  Reference  Reference  Reference  Reference  Reference  Reference  Reference  Reference  Reference  Reference  Reference  Reference  Reference  Reference  Reference  Reference  Reference  Reference  Reference  Reference  Reference  Reference  Reference  Reference  Reference  Reference  Reference | 2.33 (0.71, 7.60) 0.16  1.47 (0.92, 2.36) 0.11  1.37 (1.05, 1.80) 0.02  1.48 (1.08, 1.05) 0,02  1.17 (0.85, 1.60) 0.34  1.42 (1.01, 1.73) 0.04  1.17 (0.85, 1.60) 0.68  1.21 (0.64, 2.29) 0.57  1.51 (0.34, 6.68) 1.51  3.19 (1.15, 8.87) 0.03  1.10 (0.60, 2.02) 0.76  1.32 (0.96, 1.83) 0.09  1.45 (1.06, 1.98) 0.02  0.93 (0.53, 1.62) 0.80  1.33 (0.87, 2.03) 0.19  1.49 (1.07, 2.08) 0.02  1.27 (0.61, 2.63) 0.53  1.33 (0.63, 2.82) 0.45  1.17 (0.85, 1.60) 0.33  1.36 (1.02, 1.83) 0.04  1.30 (0.73, 2.33) 0.38  1.30 (0.86, 1.98) 0.22  1.37 (1.05, 1.79) 0.02  1.44 (0.84, 2.49) 0.19  1.32 (1.03, 1.70) 0.03  0.95 (0.68, 1.33) 0.76  1.14 (0.80, 1.61) 0.46  1.86 (1.23, 2.81) 0.003 |

BMI: Body mass index; CHD: Chronic heart disease
